# Serum Lipid Profile Constituents as Markers of Cardiovascular Morbidity in Patients on Chronic Hemodialysis

**Published:** 2007-02-07

**Authors:** Dimitrios Kirmizis, Evangelia Koutoupa, Apostolos Tsiandoulas, Aphroditi Valtopoulou, Georgios Niavis, Phani Markou, Konstantinos Barboutis

**Affiliations:** 1 Department of Nephrology, Serres General Hospital, Serres, Greece; 2 Laboratory of Immunology and Biochemistry, Serres General Hospital, Serres, Greece; 3 Dialysis Unit, Veliki Clinic, Katerini, Greece

**Keywords:** cardiovascular disease, lipids, apolipoproteins, lipoprotein (a), hemodialysis, validation

## Abstract

We designed the present case-control study in order to examine the validity of apolipoprotein (apo) A-I, B, apoB/apoA-I ratio and Lp(a) as alternative markers of cardiovascular morbidity in end-stage renal disease (ESRD) patients undergoing chronic hemodialysis (HD). Twenty-five HD patients (18 males, mean age 63, range 52–69 years) comprised the group with prevalent cardiovascular disease (CVD) and 50 HD patients (35 males, mean age 62, range 40–77 years) with non evident cardiovascular disease history constituted the second study group. Patients with CVD had significantly higher concentrations of serum apoB, apoB/apoA-I ratio and Lp(a), and lower levels of apoA-I compared to patients without incident CVD. All three parameters studied were correlated with cardiovascular morbidity, i.e. apoA-I negatively and apoB and apoB/apoA-I ratio positively (r = −0.6, *P* < 0.05; r = 0.659, *P* < 0.01; and r = 0.614, *P* < 0.01, respectively). Furthermore, logCRP exhibited as well a significant positive correlation with cardiovascular morbidity (r = 0.704, *P* < 0.001), not this being the case for Lp(a) which was not found to exhibit such a correlation (r = 0.05, *P* = NS). Among them, apoB and apoB/apoA-I ratio exhibited the characteristics most coherent to CVD. The age- and sex-adjusted OR for the presence of CVD was 2.3 and 2.0, respectively, which remained independent of any confounding effect of inflammation. In conclusion, serum apoB levels and apoB/apoA-I ratio exhibit characteristics of credible independent markers of in HD patients.

## Introduction

Serum lipid profile has long been considered to be amongst the principal cardiovascular disease risk factors in the general population as well as end-stage renal disease (ESRD) patients on chronic hemodialysis (HD). Large epidemiological and clinical studies have consistently shown that elevated serum low-density lipoprotein (LDL) cholesterol levels are associated with increased risk of cardiovascular disease (CVD) (Grundy, 2002; De Backer et al. 2003; Grundy et al. 2004). The Adult Treatment Panel III (ATP) report of the U.S. National Cholesterol Education Program (NCEP) accordingly identified elevated LDL cholesterol as the primary target of lipid-lowering therapy for reducing CVD risk (Adult Treatment Panel III 2001). Although the contribution of LDL to the development of atherosclerosis is well established, its superiority as marker of cardiovascular morbidity and mortality over other circulating constituents of lipid profile is debatable. This holds true both for the general population as well as HD patients, the later even more since there is hard evidence that some lipid constituents have a reverse prognostic significance compared to the former population (Coresh et al. 1998; Lowrie and Lew, 1990; Iseki et al. 2002). Amongst other predictors of cardiovascular outcome that have been studied over the last years, lipoprotein(a) (Lp(a)) (Tsimikas et al. 2005; Danesh et al. 2000; Cressman et al. 1992) and apolipoproteins (apo) A-I and B (Lamarche et al. 1996; St-Pierre et al. 2005; Walldius et al. 2001) have emerged as probably potent markers of cardiovascular mortality. Therefore, we designed the present case-control study in order to examine the validity of serum lipid profile components as markers of cardiovascular morbidity, study their significance as predictors of cardiovascular mortality and spot any differences between them.

## Materials and Methods

### Patients

This is a single-centre, hospital-based case-control study comprised of ESRD patients on hemodialysis. Follow-up period was 60 months. Out of a total of seventy-eight Caucasian patients that were eligible for enrolment in the study, seventy-five patients remained eligible until the end of follow-up (Tables 1 and 2). The other three patients received a renal transplant during this period and were eventually withdrawn from the study. The first study group was comprised of 25 patients with history of prevalent documented CVD during the follow-up period (18 males, mean age 63, range 52–69 years). Of these 25 patients, fifteen had a history of myocardial infarction, coronary artery bypass or angina pectoris evident clinically and on ECG, four had suffered an ischemic cerebrovascular event, evident clinically and on CT, and six had peripheral vascular disease clinically evident and documented by doppler ultrasonography and/or angiographically. At the time of enrolment into the study, no patient had any acute cardiovascular accident during the previous three months. The second patient group was comprised of 50 HD patients (35 males, mean age 62, range 40–77 years) without a history of prevalent documented CVD. Frequency matching for age and sex was performed, and a case-control ratio of 1:2 was intended. Patients with active infections or acute inflammation, liver disease, autoimmune diseases or malignancies, patients receiving antibiotics, corticosteroids or cytotoxic drugs at the time of the study as well as patients with fasting serum triglycerides >400 mg/dL (baseline measurement) were considered non-eligible for inclusion in the study. All patients had been stabilized on renal replacement therapy for ≥6 months (mean duration 65 months, range 6–210 months) and were clinically stable. Patients receiving statin therapy before their enrolment in the study were proportionately allocated in the two study groups (Table 2). All patients were receiving conventional 4-h HD, three times weekly, with bicarbonate dialysate and low-molecular-weight heparin as standard anticoagulation. Dialysis prescription was guided by a goal of achieving a value of 0.65 for the urea reduction ratio and a value of Kt/V 1.2. The above indices of adequacy of dialysis were calculated by the formula [(pre-dialysis urea)−(post-dialysis urea)/predialysis urea] and by the second-generation Daugirdas equation, respectively. Thirteen patients (17%) were active smokers. Smoking habit was defined as regular tobacco use within the previous 5 years. Systolic and diastolic arterial BP was calculated as the mean of all consecutive pre-dialysis readings during the month preceding the study start point. Mean arterial BP (MAP) was calculated as diastolic BP+ ((systolic BP-diastolic BP)/3). Diagnosis of hypertension was established by standard criteria (1999 World Health Organization, 1999). Approval of the study by the institutional review board was obtained and informed consent was obtained from each patient.

### Methods

Blood samples from HD patients were taken from a peripheral vein under overnight fasting conditions, in the morning of a midweek routine dialysis day. Serum samples were separated from clotted blood by immediate centrifugation (1500 *g* for 15 min), aliquoted and stored at −40°C until assayed. Nephelometry was used for the quantitative determination of serum high sensitivity C-reacting protein (CRP) levels. Automated latex enhanced turbidimetric immunoassay was used for the quantitative determination of Lp(a) in the serum, and immunoturbidimetric assay with the use of specific antiserum for apolipoprotein A-I and B determination. Reference value ranges for apoA-I, apoB, Lp(a) and CRP were 1.10–2.15 g/L, 0.55–1.40 g/L, <300 mg/L and 0–5.0 mg/L, respectively. All reagents used were purchased from Dade Behring (Dade Behring Inc., Deerfield, IL, USA). Serum albumin, total cholesterol, triglycerides and HDL cholesterol were determined by routine techniques using an automated analyser (Olympus AU 640, Medicon Hellas, Gerakas, Greece). LDL cholesterol was calculated using the Friedewald formula (LDL = total cholesterol–HDL–triglycerides/5).

### Statistical analysis

Data are expressed as mean ± SD and with range. Differences in categorical data were tested with the use of *X*^2^ statistic. Differences in continuous variables were assessed with *t*-test, after testing for normal distribution with the Kolmogorov-Smirnov test. Serum CRP levels followed a skewed distribution to the left and, therefore, were log-transformed before analyzed (logCRP). Correlations were tested by regression analysis or Pearson correlation analysis, as appropriate. Diagnostic accuracy of the parameters was evaluated with the determination of diagnostic sensitivity and specificity, the likelihood ratio (LR) and the receiver operating characteristics (ROC) curve of each, which enabled us to identify the best cut-off value. These cut-off points were calculated and used to transform individual continuous parameters into binary endpoints. The calculations were performed using SPSS v. 11.0.0 statistical software (SPSS, Inc, Chicago, IL, USA). A two-tailed *P*-value <0.05 was considered statistically significant.

## Results

### Mean values and distributions

The mean serum apoA-I, apoB and Lp(a) concentrations were 1.16 ± 0.15 g/L, 1.22 ± 0.2 g/L and 350 ± 160 mg/dL in males and, respectively, 1.21 ± 0.2 mg/dL, 1.21 ± 0.23 g/L and 420 ± 200 in females (*P* < 0.001 for all three variables). The mean apoB/apoA-I ratio was 1.09 ± 0.27 in men versus 1.06 ± 0.36 in females (*P* < 0.05). Serum apoA-I, apo-B and Lp(a), levels were found to follow a normal distribution, as was the apoB/apoA-I ratio. However, CRP levels followed a skewed distribution to the left and, therefore, were log-transformed before analyzed. Mean serum levels for the two study groups are shown on Table 3.

### Association of apoB levels and apoB/apoA-I ratio with cardiovascular morbidity

On correlation analysis, apolipoproteins and their ratio were found to be correlated with cardiovascular morbidity, i.e. apoA-I negatively and apoB and apoB/apoA-I ratio positively (r = −0.6, *P* < 0.05; r = 0.659, *P* < 0.01; and r = 0.614, *P* < 0.01, respectively). Furthermore, logCRP exhibited as well a significant positive correlation with cardiovascular morbidity (r = 0.704, *P* < 0.001), not this being the case for Lp(a) which was not found to exhibit such a correlation (r = 0.05, *P* = NS). None of the classic lipid parameters (total cholesterol, HDL, LDL, triglycerides) was correlated with CVD prevalence (r = −0.15, *P* = NS; r = −0.04, *P* = NS; r = 0.07, *P* = NS; and r = −0.29, *P* = NS, respectively). Moreover, apoB levels did not correlate with LDL levels (r = 0.06, *P* = NS). When the diagnostic characteristics of the tests for all molecules under study were analyzed in comparison, the molecule with the highest sensitivity, specificity and likelihood ratio for cardiovascular morbidity and AUC in RO curves was apoB, with the apoB/apoA-I ratio following in close, for values above the cut-off point of 1.26 g/L and 1.13, respectively, which were found to be the values combining both the highest sensitivity and specificity scores (Table 4). Serum apoA-I was shown to be of inferior strength as marker of cardiovascular morbidity, with likelihood ratio of 2.8.

As apoB levels and apoB/apoA-I ratio emerged to be the most intimate markers of cardiovascular morbidity in our study, we focused further on the factors affecting their association with CVD. On logistic regression analysis (Tables 5 and 6), the age- and sex-adjusted OR for the presence of CVD was 2.3 (95% confidence interval [CI], 2.0 to 2.7) and 2.0 (95% confidence interval [CI], 1.6 to 2.4) when apoB levels above 1.26 g/L and apoB/apoA-I ratio values above 1.13 were compared with values below these cut-off points, respectively. When adjustments for several patho-physiological clusters of variables were carried out, the OR of CVD associated with apoB levels above 1.26 g/L did not change appreciably after controlling for non-lipid risk factors [BMI, smoking status, hypertension, DM] (OR, 2.3; 95% CI, 1.9 to 2.7), Lp(a) (OR, 2.3; 95% CI, 1.8 to 2.8) or markers of inflammation [CRP, leukocytes] (OR, 2.2; 95% CI, 1.8 to 2.6). Alike, for apoB/apoA-I ratio values above 1.13, the OR did not change essentially after controlling for various confounders: respectively, non-lipid risk factors (OR, 2.0; 95% CI, 1.7 to 2.3), Lp(a) (OR, 2.0; 95% CI, 1.7 to 2.2) or markers of inflammation (OR, 1.9; 95% CI, 1.5 to 2.3). In all models of both variables, the OR remained meaningful and statistically significant.

## Discussion

Lipid profile constituents, such as high total cholesterol and LDL-C levels, which represent classic cardiovascular risk factors in the general population, have been repeatedly found to convey a rather inverse prognostic significance in patients on HD (Lowrie and Lew, 1990; Iseki et al. 2002). In the prospective study by Liu et al. it became clear that this adverse association between total cholesterol levels and mortality is due to the cholesterol-lowering effect of systemic inflammation and malnutrition rather than to a speculated protective effect of high cholesterol levels (Liu et al. 2004). The search for more accurate markers amongst the lipid profile has brought to the surface serum apolipoproteins, as probably better markers of the balance between preatherogenic and atherogenic lipoproteins and predictors of cardiovascular risk (Shoji et al. 2001; Walldius et al. 2004; Chan and Watts, 2006). Their measurement has several advantages over HDL-C and LDL-C: fasting samples are not required, they are a better index of the adequacy of statin therapy and their measurement techniques are standardized (Chan and Watts, 2006). The present study was set up to determine and compare the validity of the serum apolipoproteins, their ratio and serum Lp(a) as markers of cardiovascular morbidity in patients on HD. ApoA-I is the major apolipoprotein constituent of the anti-atherogenic high-density lipoproteins (HDL). Levels of apoA-I are strongly associated with those of HDL cholesterol. ApoA-I is critically involved in removing excess cholesterol from tissues and incorporating it into HDL for reverse transport, either directly or indirectly via LDL to the liver. ApoB stands for apolipoprotein B-100 and is the chief protein component constituent of the atherogenic very-low-density lipoprotein (VLDL), of intermediate-density lipoprotein (IDL) and of LDL particles, each particle including one apoB molecule. Consequently, plasma apoB levels reflect the total numbers of atherogenic particles. In humans, VLDL particles carry endogenously synthesized triglyceride from the liver into plasma, where they undergo lipolysis to IDL by the action of lipoprotein lipase. IDL is lipolysed by hepatic lipase, converting to LDL, or taken up by the liver via the LDL receptor. ApoB is also essential for the binding of LDL particles to the LDL receptor for cellular uptake and degradation of LDL particles (Simon et al. 2005). Lp(a) is an atherogenic lipoprotein and consists of an LDL-cholesterol particle covalently bonded to apoA. Lp(a) is strongly and negatively associated with apoA isoform size (Marcovina et al. 1995) and is elevated in HD patients (Kronenberg et al. 1995), suggesting that Lp(a) level or small apoA may account for a portion of the increased atherosclerotic CVD or mortality in ESRD patients. Several large epidemiologic studies have found that apolipoproteins, especially apoB or the ratio of apoB to apoA-I, are superior to classic lipid profile constituents (i.e. total, HDL and/or LDL cholesterol) in predicting coronary heart disease risk/myocardial infarction (Lamarche et al. 1996; St-Pierre et al. 2005; Walldius et al. 2001; Talmud et al. 2002; Meisinger et al. 2005; Jiang et al. 2004; Ridker et al. 2005; Shai et al. 2004; Moss et al. 1999; Yusuf et al. 2004) in the general population. A rather clear advantage of these two indices has been indicated as well by studies regarding treatment benefits and prediction of subsequent risk for acute coronary event in the general population (Gotto et al. 2000; Simes et al. 2002; Pedersen et al. 1998; Roeters van Lennep et al. 2000). In ESRD patients, there are scarce reports for the value of apolipoproteins as markers of cardiovascular mortality and morbidity (Kronenberg et al. 1995; Hahn et al. 1983; Shoji et al. 1997; Lee et al. 2002). In our study, patients with prominent CVD had elevated serum apoB and Lp(a) levels and apoB/apoA-I ratio values, as well as lower serum levels of apoA-I compared to patients without prevalent CVD. The former had also significantly higher concentrations of serum CRP and significantly lower concentrations of serum total cholesterol, HDL, LDL, triglycerides and albumin compared to the later. These results confirmed earlier observations that, among dialysis patients, cholesterol and LDL levels are inversely rather than directly associated with mortality rates (Coresh et al. 1998; Lowrie and Lew, 1990; Iseki et al. 2002). In our retrospective study, classic lipid levels fail to demonstrate any correlation with CVD prevalence. On the contrary, apolipoproteins and their ratio exhibited a consistent correlation with cardiovascular morbidity. Between the parameters studied, apoB exhibited the characteristics (ROC curve, area under the curve, LRs) most coherent with associated cardiovascular morbidity, with the apoB/apoA-I ratio following in close. Both parameters were found to be strongly associated with CVD, independently of other potential confounders (traditional non-lipid risk factors, inflammation markers and Lp(a)). Quite different results, however, have been reported in dialysis patients in a two-year survival study by Hocher et al. (Hocher et al. 2003), who have found apoA-I or apoB not to be significantly associated with cardiovascular mortality. However, cardiovascular morbidity was not examined in that study and the probable confounding effect of other factors might have obscured the probable association of apolipoproteins with CVD. Our results are in line with the findings of Simon et al. (Simon et al. 2005), who compared the validity of plasma apoB, non-HDL cholesterol and LDL-C in predicting cardiovascular risk, and subclinical atherosclerosis. These authors reported that apoB was consistently a stronger predictor of coronary heart disease risk, as well as peripheral and coronary atherosclerosis, than either non-HDL cholesterol or LDL-C. Moreover, it is interesting that other investigators (Schaefer et al. 1994) concluded that plasma apoB values ≥1.25 g/L may be associated with an increased risk for CHD in the general population, a cut-off value which is explicitly close to the value that came up to us (1.26 g/L). Despite the fact that our measurements were done in serum, a discrepancy of no more than 3% from plasma values is expected (Abbott et al. 1983). In the existing literature, there is no hard evidence as yet regarding the possible effects of inflammation on apoB levels or the association between apoB and coronary artery disease. In our study no confounding effect of markers of inflammation (leukocytes, CRP) on apoB levels was established. One cannot discriminate, however, from the data of our study whether there is absolute lack of an effect or whether apoB levels remain eventually elevated due to counter-acting effects of overproduction of apoB, which is known to occur in HD patients (Chan et al. 1989), and a probable apoB-lowering effect of systemic inflammation and malnutrition. This seeming lack or counter-balance of the effect of inflammation on serum apoB levels probably represent the mechanism underlying the superior predicting significance of apoB and apoB/apoA-I ratio for CVD, compared to total cholesterol and LDL-C levels, which have been found consistently to be affected essentially by systemic inflammation in ESRD patients, and which were found not to be a reliable correlate of cardiovascular morbidity in our study. Moreover, although in many conditions LDL cholesterol and apoB levels increase in parallel (e.g. familial hypercholesterolaemia), in ESRD patients, due to hypertriglyceridemia, malnutrition or metabolic disturbances, LDL-C levels are often phenomenically ‘normal,’ as in our study, yet apoB levels are rather elevated due to its overproduction and this results in the formation of a small, dense form of LDL (pattern B phenotype). This phenotype is considered to be of particular importance in the pathogenesis of atherosclerosis, since these LDL bind less avidly to the LDL receptor which mediates its endocytosis in the liver or peripheral tissues (Liu and Rosner, 2006). This is a probable cause that helps apoB retain their credibility as a marker of dyslipidemia as much as treatment target for statin therapy, since they ‘represent’ these atherogenic small, dense LDL. These advantages render apoB measurement a valuable adjunct measurement to the existing routine lipid profile in ESRD patients.

There are several restrictions of the present study. First, this is a retrospective study and, as such, its results must be confirmed by large prospective studies. Second, the clinical description of non-CVD patients is a rather rough one, and our results should be confirmed by studies with the use of more invasive methods, such as myocardial perfusion scintigraphy, electron-beam CT or coronary angiography. Moreover, the conclusions that have been reached from this study of cardiovascular morbidity cannot be extrapolated to pre-clinical or latent cardiovascular pathology. The validity of the studied parameters as markers of atherosclerosis or early cardiovascular disease should be further addressed by larger studies with the use of delicate methods, such as the Doppler measurement of carotid intima-media thickness or plaque-score.

## Conclusions

This study showed that among the constituents of the lipid profile that were studied, apoB as well as apoB/apoA-I ratio appears to be the markers most closely related to CVD in ESRD patients. Both were found to be essentially independent markers of CVD in dialysis patients, not affected by the state of chronic inflammation of this population. As they are more stable and practical molecules to measure, apoB measurement and/or apoB/apoA-I ratio calculation should be probably introduced into the gamut of risk predictors for atherosclerotic disease in HD patients, provided that larger prospective studies confirm the preliminary results of the present study.

## Figures and Tables

**Table 1:** Demographic and clinical characteristics of the patients.

	HD patients
Males/females (*n*, %)	53/22, 70/30
Age (mean, range) (years)	62 (40–77)
Primary disease	
• Interstitial nephritis (*n*, %)	21, 28
• Glomerulonephritis (*n*, %)	22, 29
• Diabetic nephropathy (*n*, %)	14, 19
• Vascular diseases (*n*, %)	5, 7
• Polycystic kidney disease (*n*, %)	5, 7
• Alport’s syndrome (n, %)	5, 7
• Unknown (*n*, %)	3, 4
Duration of haemodialysis (mean, range) (months)	65 (6–210)
Kt/V	1.19 ± 0.24
BMI (mean, range) (kg/m^2^)	24.5 (19.5–25.6)
Cardiovascular disease (*n*, %)	25, 33
• Coronary heart disease (*n*, %)	15, 20
• Cerebrovascular disease (*n*, %)	4, 5
• Peripheral vascular disease (*n*, %)	6, 8

**Table 2:** Demographic data and clinical characteristics of the patient groups.

	Prevalent CVD (*n* = 25)	Not prevalent CVD (*n* = 50)	*P* value
Age (mean, range) (years)	63 (52–69)	62 (40–77)	0.47
HD duration (mean, range) (months)	63 (17–210)	67 (6–210)	0.03
Kt/V	1.20 ± 0.3	1.19 ± 0.6	0.6
BMI (mean, range) (kg/m^2^)	24.0 (19.5–30.6)	24.1 (20.0–28.4)	0.13
Pre-dialysis systolic BP (mmHg)	143 ± 18	139 ± 16	0.03
Pre-dialysis diastolic BP (mmHg)	83 ± 8	85 ± 6	0.05
Pre-dialysis MAP (mmHg)	103 ± 11	102.7 ± 9.2	0.072
Diabetes mellitus (*n*, %)	4,16	10, 20	0.8
Active smoking (*n*, %)	5, 20	8, 16	0.08
Dialyzer membrane (*n*, %)			
• Hemophan	15, 60	29, 58	0.76
• Polysulfone	8, 32	15, 30	0.4
• Polyacylonitryle	2, 8	6, 12	0.3
Erythropoetin (iv) treated (*n*, %)			
• Epoetin alfa	12, 48	23, 46	0.42
• Epoetin beta	4, 16	10, 20	0.06
• Darbepoetin alfa	5, 20	13, 26	0.05
Antihypertensive medication used:			
• CCBs (*n*, %)	18, 72	35, 70	0.26
• CEIs or ARBs (*n*, %)	7, 28	12, 24	0.3
• B-blockers (*n*, %)	3, 12	5, 10	0.35
Lipid lowering therapy used:			
• Statins	3	6	0.8
• Fibrates	0	0	
• Other	0	0	
Antiplatelet medication used:			
• Ticlopidine	12, 48	22, 44	0.07
• Aspirin	3, 12	5, 10	0.2
• Clopidogrel	6, 24	6, 12	0.03

Abbreviations: CCBs: Ca^++^-channel inhibitors; CEIs: converting enzyme inhibitors; ARBs: angiotensin-II receptor blockers.

**Table 3:** Laboratory examinations of the study groups.

	Not prevalent CVD (*n* = 50)	Prevalent CVD(*n* = 25)
ApoA-I (g/L)	1.26 ± 0.2†	1.04 ± 0.1†
ApoB (g/L)	1.13 ± 0.18†	1.39 ± 0.14†
ApoB/apoA-I	0.92 ± 0.26†	1.35 ± 0.17†
Lp(a) (mg/L)	359 ± 275†	460 ± 260†
CRP (mg/L)	3.5 ± 1.8†	7.8 ± 4.0†
Total cholesterol (mmol/L)	4.89 ± 1.11†	3.80 ± 0.98†
LDL-C (mmol/L)	2.90 ± 0.98†	2.12 ± 0.65†
HDL-C (mmol/L)	1.01 ± 0.26†	0.93 ± 0.18†
Triglycerides (mmol/L)	2.15 ± 1.14†	1.63 ± 0.73†
Hemoglobin (g/L)	114 ± 11	115 ± 12
White blood cell count(×10^9^/L)	7.2 ± 1.6	7.5 ± 1.7
Platelet count (×10^9^/L)	276 ± 77	277 ± 88
Albumin (g/L)	40 ± 3	39 ± 4

† *P* < 0.05, when patients with prevalent CVD were compared to patients without prevalent CVD.

**Table 4:** Test characteristics of the parameters, for cutoff points with the optimal sensitivity and specificity scores.

	ApoA-I	ApoB	ApoB/apoA-I
Cut-off point	1.025 g/L	1.26 g/L	1.13
AUC†	0.14	0.894	0.878
95% CI	0.132–0.142	0.889–0.898	0.874–0.883
Sensitivity (%)	55	100	100
Specificity (%)	15	75	77
LR	2.8	17.1	9.6

† Area under the curve

**Table 5:** Odds ratio of CVD associated with apoB levels above 1.26 g/L, after various adjustments.

Model No.	Variable	Odds ratio	95% CI
1	Age and sex	2.3	2.0–2.7
2	Non-lipid risk factors†	2.3	1.9–2.7
3	Lp(a)	2.3	1.8–2.8
4	CRP, leukocytes	2.2	1.8–2.6

All models are adjusted for age and sex.

† BMI, smoking status, hypertension, DM.

**Table 6:** Odds ratio of CVD associated with apoB/apoA-I ratio values above 1.13, after various adjustments.

Model No.	Variable	Odds ratio	95% CI
1	Age and sex	2.0	1.6–2.4
2	Non-lipid risk factors†	2.0	1.7–2.3
3	Lp(a)	2.0	1.7–2.2
4	CRP, leukocytes	1.9	1.5–2.3

All models are adjusted for age and sex.

† BMI, smoking status, hypertension, DM.
